# Investigation of the spatial and temporal variation of soil salinity using Google Earth Engine: a case study at Werigan–Kuqa Oasis, West China

**DOI:** 10.1038/s41598-023-27760-8

**Published:** 2023-02-16

**Authors:** Shilong Ma, Baozhong He, Boqiang Xie, Xiangyu Ge, Lijing Han

**Affiliations:** 1 College of Geography and Remote Sensing Sciences, Xinjiang University, No. 777 Huarui Street, Xinjiang 830017 Urumqi, China; 2 Xinjiang Key Laboratory of Oasis Ecology, Xinjiang University, 830017 Urumqi, China; 3 Key Laboratory of Smart City and Environment Modelling of Higher Education Institute, Xinjiang University, 830017 Urumqi, China

**Keywords:** Environmental sciences, Natural hazards

## Abstract

Large-scale soil salinity surveys are time-costly and labor-intensive, and it is also more difficult to investigate historical salinity, while in arid and semi-arid regions, the investigation of the spatial and temporal characteristics of salinity can provide a scientific basis for the scientific prevention of salinity, With this objective, this study uses multi-source data combined with ensemble learning and Google Earth Engine to build a monitoring model to observe the evolution of salinization in the Werigan–Kuqa River Oasis from 1996 to 2021 and to analyze the driving factors. In this experiment, three ensemble learning models, Random Forest (RF), Extreme Gradient Boosting (XGBoost), and Light Gradient Boosting Machine (LightGBM), were established using data collected in the field for different years and some environmental variables, After the accuracy validation of the model, XGBoost had the highest accuracy of salinity prediction in this study area, with RMSE of 17.62 dS m^−1^, R^2^ of 0.73 and RPIQ of 2.45 in the test set. In this experiment, after Spearman correlation analysis of soil Electrical Conductivity (EC) with environmental variables, we found that the near-infrared band in the original band, the DEM in the topographic factor, the vegetation index based on remote sensing, and the salinity index soil EC had a strong correlation. The spatial distribution of salinization is generally characterized by good in the west and north and severe in the east and south. Non-salinization, light salinization, and moderate salinization gradually expanded southward and eastward from the interior of the western oasis over 25 years. Severe and very severe salinization gradually shifted from the northern edge of the oasis to the eastern and southeastern desert areas during the 25 years. The saline soils with the highest salinity class were distributed in most of the desert areas in the eastern part of the Werigan–Kuqa Oasis study area as well as in smaller areas in the west in 1996, shrinking in size and characterized by a discontinuous distribution by 2021. In terms of area change, the non-salinized area increased from 198.25 in 1996 to 1682.47 km^2^ in 2021. The area of saline soil with the highest salinization level decreased from 5708.77 in 1996 to 2246.87 km^2^ in 2021. overall, the overall salinization of the Werigan–Kuqa Oasis improved.

## Introduction

Soil salinization has become one of the threats to global agricultural systems^1^, and it is expected that with climate change, the impact of salinization will be wider and the degree of harm will increase, in addition, the formation mechanism of salinization is complex^2^. For regulating salinization and preventing soil degradation, it is crucial to comprehend the characteristics of salinization's spatial and temporal distribution and its evolutionary patterns^3^.

Traditional laboratory analysis for soil salinity monitoring is time-consuming and labor-intensive, and because salinity changes widely across space and time, it is challenging to precisely characterize the geographical distribution of salinity and its evolutionary patterns^4^. Digital mapping has made a splash in the field of soil science, thanks to the advancement of computer hardware and software, as well as the creation of geographic information systems, global positioning systems, remote or proximity sensors, and digital elevation models that have generated huge volumes of data^5^, The use of remote sensing techniques to detect salinity has increased in importance with the emergence of remote sensing satellites. Microwave and multitemporal optical remote sensing are efficient methods for identifying surface salinity parameters^6^.

Various salinity indices have been constructed for modeling and prediction using the rich waveband information of optical satellites^7,8^. As in the instance of Khan et al.^9^ who utilized salinity indices (SI) to categorize and analyze salinity-prone terrain, remote sensing-based salinity indices can instantly respond to the salinity status of the surface in places where it is barren or sparsely vegetated. Due to the influence of other elements including soil moisture, vegetation cover, and data collection time, it is extremely challenging to obtain pure saline spectral information in natural situations. Because salt-tolerant plants thrive in arid and semi-arid climates, vegetation index is employed as an Indirect indicator for salinity^10^. Many salinity prediction studies, such as Ramos, et al.^7^ used the Canopy Response Salinity Index (CRSI), Enhanced Vegetation Index (EVI), and Normalized Difference Vegetation Index (NDVI) to assess salinity in the field; other indices widely used for salinity monitoring are Soil Adjust Vegetation Index (SAVI), Ratio Vegetation Index (RVI), and Divergence Vegetation Index (DVI), and Green Vegetation Index (GVI)^11,12^.

The formation of soil salinity is highly nonlinearly related to many environmental factors, and machine learning algorithms are popular in the field of salinity research using their efficient data mining capabilities^13,14^. It has been difficult to choose the optimal model for a specific area when digitally mapping soils, but machine learning has been demonstrated to perform better than conventional statistical models at accurately predicting salinity^15,16^. The performance of various machine learning algorithms has also been compared with linear regression models and among machine learning algorithms for salting inversion analysis, including Multi-Layer Perceptron-Artificial Neural Network (MLP-ANN), Multivariate Adaptive Regression Splines (MARS), Classification and Regression Tree (CART), support vector regression (SVR), and RF. With the maturation of the ensemble learning method, it is frequently employed in picture classification research^17^, nevertheless, it is not commonly used in soil salinity prediction studies. To assess the geographical variability of soil salinity and alkalinity in agricultural regions impacted by salinity, several researchers have employed random forests, with satisfactory results^18^. Recent studies that forecast salinity have employed XGBoost^19,20^, while other ensemble learning techniques, including light gradient boosting machine, have seldom ever been published in the field of salinity research LightGBM^21^. Therefore, in this study, three ensemble learning models were applied to the prediction and mapping of salinity to evaluate their potential application in salinity monitoring efforts. Long-term salinity monitoring in arid and semi-arid areas is essential because it can adequately address local human-land linkages and serve as a guide for salinity control. The enormous volume of data makes the information extraction procedure in multi-temporal remote sensing challenging. Advantageously, Google Earth Engine offers a powerful data processing platform that includes a variety of geographical data, including various types of remote sensing data^22^. The spatial and spectral resolution of multispectral remote sensing is well suited for salinity monitoring due to its large coverage and ease of acquisition^6,23^. In this study, Landsat5 TM and Landsat OLI satellites were selected as the remote sensing data sources for this study because of the need to predict the salinity distribution in the inversion epoch and because of the good performance of Landsat satellites in salinity monitoring^24,25^.

In this study, four years of experimental data were aggregated to make the prediction model more stable and to produce more accurate information on the spatial distribution of salinization. The specific objectives of this study were: (1) Evaluating the predictive power of RF, XGBoost, and LightGBM in ensemble learning for soil conductivity (2) Digital mapping of salinity distribution in 1996, 2006, 2017, and 2021 based on remote sensing data using an optimal prediction model; (3) The spatial and temporal variable features of salinization in Werigan–Kuqa Oasis during the last 25 years; (4) Discuss the effects of arable land expansion and land remediation on salinity.

## Materials and methods

### Study area

**Figure 1:**
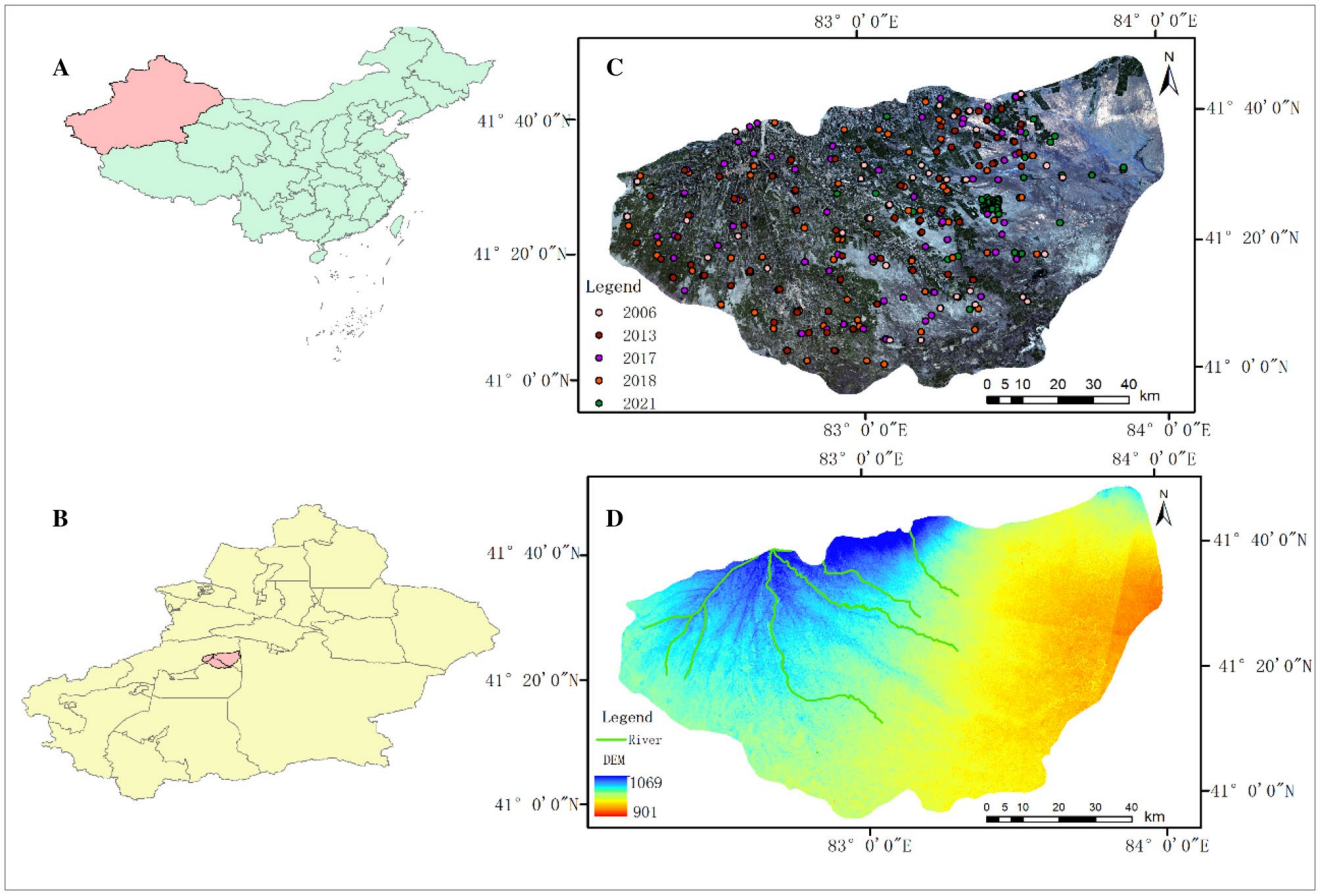
Figure (**A**) shows the location of Xinjiang, Figure (**B**) shows the location of the study area in Xinjiang, Figure (**C**) shows the distribution of sampling sites in the study area in different years, and figure (**D**) is the elevation of the study area.

The area of study is the Werigan–Kuqa River Oasis (also known as the Werigan–Kuqa Oasis), which is situated at an altitude of 901–1069 m above sea level in the north-central Tarim Basin of the Xinjiang Uygur Autonomous Region. It has an area of around 9769.76 km^2^. The Werigan–Kuqa Oasis features a typical warm-temperate continental dry climate due to its deep interior location and distance from the sea, with average annual precipitation and evaporation of 70 and 1100 mm, respectively, and a high evapotranspiration ratio of 16:1. The research region mostly consists of desert, agriculture, grassland, and woodlands, with salt- and drought-tolerant plants flourishing in the desert. Werigan–Kuqa Oasis is generally flat, with a high water table, a long dry season, and strong evaporation. In this context, salts can easily accumulate on the surface, so the area chosen as the study area is representative and has great significance for the improvement of the ecological environment and the development of agricultural production (Fig. 1).

### Sample collection and survey

Field sampling and surveys of the Werigan–Kuqa Oasis are conducted annually, with most of the sampling taking place in July each year. The location of sampling points as well as the number of sampling points were determined by combining existing digital soil maps (salinity maps, soil type, soil texture) and land use/cover types, while sampling strategies were changed based on field observations from the previous year to take into account changes from year to year (Fig. 1). The location of each sampling point is recorded using a portable GPS, and the soil samples are packed in (approximately 500 g) transparent sealed bags for the next step of laboratory analysis. In this study, 4 years of soil surface (0–10 cm) electrical conductivity (EC) data were summarized and screened. The sampling times in the field were July 2006, with 36 samples; July 2017, with 84 samples; July 2018, with 75 samples, and June 2021, with 63 samples. All samples underwent air drying, grinding, homogenization, and sieving at a 0.15 mm size. For every 20 g of soil, add 100 ml of distilled water, mix thoroughly for 30 min, and then leave for 24 h. At room temperature of 25 °C, the soil conductivity was measured using a digital multiparameter measuring system (Multi 3420 Set B, WTW GmbH, Germany) fitted with a composite electrode (TetraCon 925)^26^.

### Environmental variables

The key to the selection of environmental variables is that the covariates must respond to the nature of soil formation, climate, biology and landscape type, etc. According to the SCORPAN framework (S is for soil, C is for climate, O is for organisms, R is for relief, P is for parent material, A is for age, and N is for space.)^5^, a series of environmental factors were selected, including each of the original bands of Landsat5 TM and Landsat8 OLI, various indices derived from remote sensing (vegetation index, salinity index), elevation data and their derived indices (e.g. terrain moisture index, TWI).

#### Remote sensing-based environment variables

In this study, the remote sensing-based index extraction was done in the Google Earth Engine cloud platform. The Landsat5 TM image of July 22, 2006, and Landsat8 OLI images of July 4, 2017, July 23, 2018, and July 15, 2021, are selected, which matched the sampling time, were selected to have less than 10% cloudiness. The remote sensing-based environmental variables include 6 raw bands, 12 vegetation indices, 9 salinity indices, 1 carbonate index, and 1 brightness index (Table 1).

**Table 1:** Auxiliary data based on remote sensing.

Auxiliary	Index	Acronym	Formula	Reference
Vegetation indices	Normalized difference vegetation index	NDVI	(NIR−R)/(NIR + R)	^27^
	Generalized difference vegetation index	GDVI	(NI R^2^−R^2^)/(NI R^2^ + R^2^)	^27^
	Normalized difference vegetation index	GNDVI	(NIR−G)/(NIR + G)	^28^
	Green ratio vegetation index	GRVI	NIR/(G−1)	^29^
	Optimizied soil adjusted vegetation index	OSAVI	(NIR−R)/(NIR + R + θ)	^30^
	Ratio vegetation index	RVI	NIR/R	^31^
	Soil adjusted vegetation index	SAVI	((NIR−R)/(NIR + R + L))*(1 + L)	^32^
	Brightness index	BRI	(G^2^ + R^2^)^0.5^	^33^
	Carbonate index	CAEX	B/G	^33^
	Canopy response salinity	CRSI	(((NIR*R)−(G*B))/((NIR*R) + (G*B)))^0.5^	^34^
	Difference vegetation index	DVI	NIR−R	^35^
	Enhanced vegetation index	EVI	2.5*(NIR−R)/(NIR + 6R−7.5B + 1)	^36^
	Green atmospherically resistant vegetation index	GARI	(NIR−(G + y*(B-R)))/(NIR + (G + y*(B + R)))	^37^
	extented EVI	EEVI	2.5*(NIR + SWIR1−R)/(NIR + SWIR1 + 6*R−7.5*B + 1)	^38^
Soil-related indices	Salinity index	SIT	(R/NIR)*100	^39^
	Salinity index	SI	(R−NIR)/(R + NIR)	^39^
	Salinity index	SI1	(R*G)^0.5^	^39^
	Salinity index	SI2	(NIR^2^ + R^2^ + G2)^0.5^	^39^
	Salinity index	SI3	(R^2^ + G2)^0.5^	^39^
	Salinity index	SI4	(R*NIR)/G	^40^
	Salinity Ratio index	SAIO	(R−NIR)/(G + NIR)	^33^
	Salinity index	SIA	(B/R)	^39^
	Salinity index	SIB	(B−R)/(B + R)	^33^

#### Terrain attributes

In this study, 11 topographic indices were generated using 30 m resolution DEM data from the Geospatial Data Cloud (http://www.gscloud.cn/), clipped, and stitched together using SAGA GIS software (Table 2). The results of Vermeulen and Van Niekerk^41^ showed that the use of elevation data and its derived topographic indices as geostatistical and machine learning input variables have a great potential for salinity prediction to monitoring salt accumulation in irrigated areas.

**Table 2:** Terrain attributes.

Auxiliary	Index	Acronym	Reference
Dem derivatives	Aspect	ASP	SAGA GIS
	Convergence index	CI	
	LS-factor	LSF	
	Relative slope position	RSP	
	Slope	Slope	
	Topographic wetness index	TWI	
	Valley depth	VD	
	DEM	DEM	
	Channel network distance	CND	

### Model framework

#### Random forest

Random Forest, developed by Breiman^42^, is a popular ensemble learning algorithm based on tree-based bagging (bootstrap aggregation)^43^, which has the advantage of having nonlinear mining capabilities, data distribution that does not need to conform to any assumptions, handling both rank and continuous variables, preventing overfitting, fast training, and quantitative description of the contribution of variables. RF is a bagging improvement that enhances variable selection^44^, Instead of selecting the optimal split among all characteristics at each node, RF randomly picks a subset of features to decide the split, this makes RF more resilient to noise and less prone to overfitting. In addition, RF can handle outliers very well^45^. The number of trees and predictor variables that the random forest model allows the decision tree to grow as large as it can without being trimmed is its critical factor. The primary hyperparameters modified in this study are the number of trees in the forest and the number of features thought to divide at each leaf node^46^. In this work, we used the open-source machine learning package Scikit-learn to create an RF mode^47^.

#### Extreme gradient boosting

Extreme Gradient Boosting (XGBoost) is a popular boosting-based ensemble machine learning algorithm^48^, this algorithm was used in the Kaggle signal recognition competition and has attracted a lot of attention for its outstanding efficiency and high prediction accuracy^49^. Boosting, in contrast to bagging, is an iterative method that successively adds new trees to the integration, and samples erroneously predicted by the prior tree are given higher weights in the succeeding trees. Thanks to numerous significant systematic and algorithmic enhancements, the gradient boosting framework is implemented effectively and flexibly in XGBoost^49,50^. The number of gradients boosting trees (n_ estimators), learning rate (eta), maximum depth of the tree (max_depth), and column per level of the subsample ratio are some of the important hyperparameters that are tuned by XGBoost. To train XGBoost models, the open-source Scikit-Learn software is utilized.

#### Light gradient boosting machine

Light Gradient Boosting Machine (LightGBM) is a framework that implements the idea of GBDT (Gradient Boosting Decision Tree) algorithm^51^, a boosting decision tree tool open-sourced by the Microsoft DMTK team, which has fast training speed and less memory usage, which greatly speeds up the training and also has better model accuracy. LightGBM performs the following optimizations on the traditional GBDT algorithm: Gradient-based One-Side Sampling (GOSS) and Exclusive Feature Bunding (EFB)^51^. GOSS is a subsampling technique used to create training sets to build the base tree in the integration, select data with larger gradients from the sample to increase their contribution to the computed Information gain, and EFB merges certain data features to reduce the data dimensionality^52^. Generally, the prediction accuracy is significantly influenced by the hyperparameters^53^. So, before employing LightGBM, we need the first figure out how many and how widely its hyperparameters may vary. The number of Leaves, Learning Rate, and Maximum Depth is the important factors.

For this experiment, the above three models were done in the Spyder platform based on the Python 3.9.7 programming language.

### Model parameter optimization

The efficacy of the model application depends on the choice of model parameters. In the fields of statistical analysis and machine learning, the K-Fold cross-validation method is frequently used to assess the generalizability of models. The grid search method is an exhaustive search method that specifies the values of the parameters, it is carried out by Scikit-GridSearchCV, learn's which arranges and combines the possible values of each parameter, lists all combinations that could exist, and performs cross-validation to optimize the estimation function's parameters in order to obtain the best learning algorithm^54^. The minimum value of Root Mean Square Error (RMSE) is used as the criterion for the selection of model parameters, In this experiment, it is assumed that the value of K is 5, as follows:

1. Divide the dataset into the training set, test set, and K-fold division of the training set data.
2. Determine the range of each parameter of the model, taking a random forest as an example, and determine the number of decision trees m as well as the depth h. The combination of parameters is the cross nodes of a two-dimensional grid with m and has horizontal and vertical axes.
3. Choose any K-1 data from the training set, choose a set of cross-node parameters, create one decision tree using a sample of all the K-1 data, forecast the final 1 data, and compute the average root mean square error of all trees on the final 1 training sample.
4. Repeat the above two steps until you have traversed K-1 copies of the data.
5. Iterate through the parameter combinations of all crossover nodes of the grid. 6.
6. Steps 3 to 5 are repeated, using cross-validation to calculate the performance of the model in the test dataset. (Table 3) shows the combination of model parameters optimized by grid search.

**Table 3:** Combination of parameters for different models.

	n_estimators	max_depth	learning_rate	Subsample	colsample_bytree
RF	10	10	Null	Null	Null
XGBoost	43	4	0.1	0.5	0.9
LightGBM	22	4	0.2	0.5	0.9

### Evaluation of prediction accuracy

In this research, the coefficient of determination R^2^, the root mean square error(RMSE), and the performance to interquartile distance(RPIQ) are used to assess the performance of RF, XGBoost, and Lightgbm. The closely R^2^ is to 1, the more accurate models are fitted. The closer the number is to 0, the smaller the difference between the measured value and the predicted value of the model, and the greater the model's ability to forecast the future. The value of RMSE is inversely related to the accuracy of the model. RPIQ is the interquartile range to RMSE ratio, and the interquartile range is the difference between 75 and 25% of the sample values. It is commonly accepted that RPIQ < 1.7 implies low model prediction dependability, 1.7 ≤ RPIQ ≤ 2.2 suggests somewhat balanced prediction ability, and RPIQ ≥ 2.2 indicates highly strong prediction ability. RPIQ is a more reasonable and objective measure when compared to the Ratio of Performance to Deviation (RPD), especially for soil samples with an unusual distribution^55,56^. Equations (1)–(3) show the expression of these model evaluation metrics:

1 $$R^{2} = \frac{{\sum\limits_{i = 1}^{n} {\left( {X_{i}^{*} - Y_{i}^{*} } \right)^{2} } }}{{\sum\limits_{i = 1}^{n} {\left( {X_{i} - Y_{i} } \right)^{2} } }}$$

2 $$RMSE = \sqrt {\frac{1}{n}\sum\limits_{i = 1}^{n} {(X_{i} - Y_{i} )^{2} } }$$

3 $$RPIQ = \frac{\Delta Q}{{RMSE}}$$

where *N* is the number of samples, *X*_*i*_ is the measured EC value, *Y*_*i*_ is the calculated value, *X*_i_^*^ is the mean measured EC value, *Y*_i_^*^ is the estimated soil EC value, *SD* represents the standard deviation, and *ΔQ* is the interquartile distance (IQR), which is the difference between the upper quartile (*Q3*) and the lower quartile (*Q1*).

### Soil EC prediction and mapping for different years

**Figure 2:**
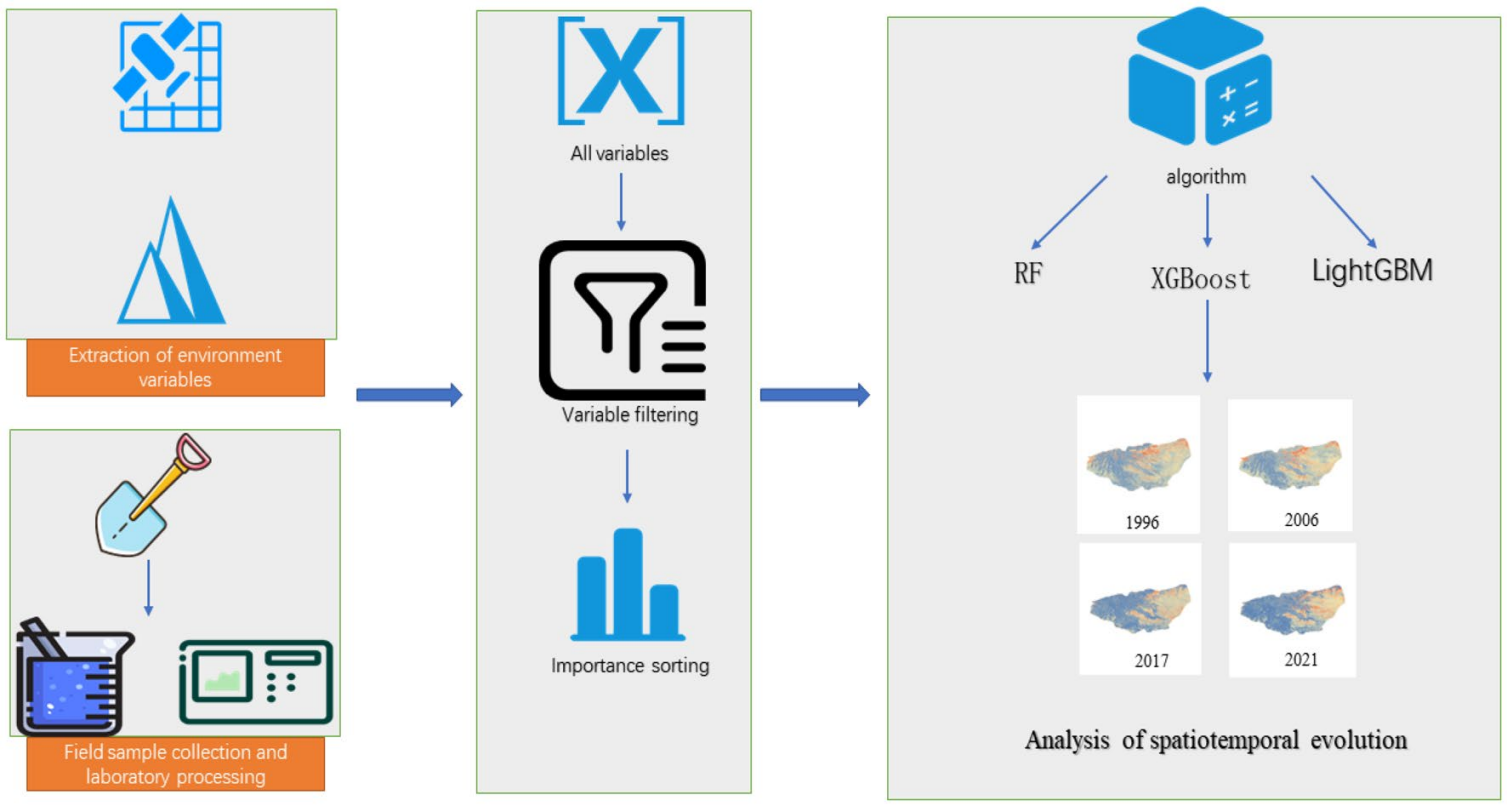
Flow chart.

The flow of this experiment is shown in (Fig. 2). The Google Earth Engine cloud platform was used to calculate and obtain the remote sensing-based environmental variables corresponding to the sampling time to establish a soil EC prediction model. Since the sampling time is mainly concentrated in July, based on the optimal model, the spatial distribution maps of soil EC in July of each year in 1996, 2006, 2017, and 2021 are obtained (the remote sensing data of June 24 is chosen because the remote sensing Image of July 1996 Is too cloudy to meet the mapping requirements), and this step is done by using the Spyder development environment with the help of GDAL, Pandas and other libraries to complete the mapping.

## Results

### Soil EC descriptive statistics

In this experiment, the final data of 258 soil EC samples were obtained after the outliers were removed from the sample data. Following statistical analysis, the soil's electrical conductivity (EC) minimum, maximum, mean, standard deviation, coefficient of variation, kurtosis, and skewness were determined (Table 4).

**Table 4:** Soil EC descriptive statistics.

EC sample data	Max(dS m^−1^)	Min(dS m^−1^)	Mean(dS m^−1^)	Std.D(dS m^−1^)	CV (%)	Kurtosis	Skewness
Whole data(n = 258)	143.4	0.079	27.9	33.2	1.19	1.15	1.37

Soil EC values in the Werigan–Kuqa Oasis ranged from 0.079 dS m^−1^ to 143.4 dS m^−1^, showing that the samples had a high span. The skewness of 1.37 is much higher than 0, which indicates that the sample data do not obey a normal distribution. The standard deviation was 33.2 dS m^−1^ and the coefficient of variation was 1.19, which is greater than 1, thus belonging to strong variability, which is consistent with the study of Wang, et al.^40^, showing the high spatial variability of soil EC values in the Werigan–Kuqa Oasis area.

### Correaltion analysis

In modeling soil salinity monitoring, not all environmental variables are involved in modeling and there are differences in their contribution to EC prediction^40^, therefore, it is necessary to screen the environmental variables. Based on the statistical analysis of the sample EC values, the skewness was 1.47 (Table 4), so Spearman correlation analysis was used in the analysis of the relationship between environmental variables and soil EC values. In this study, 38 environmental variables (original band, vegetation index, salinity index, topography index, etc.) were initially selected, and after Spearman correlation analysis, 31 environmental variables were selected and the remaining relevant variables were not significantly correlated (Table 5).

**Table 5:** The correlation between the variables and soil EC(0–10 cm).

Factors	R	Factors	R	Factors	R
EEVI	− .219**	SI	.531**	BRI	.315**
Green	.246**	SI1	.320**	CAEX	.612**
Nir	− .610**	SI2	− .308**	CRSI	− .506**
Red	.372**	SI3	.324**	DVI	− .572**
Swir1	.300**	GDVI	− .541**	EVI	− .596**
Swir2	.423**	GNDVI	− .468**	GARI	− .626**
SI4	− .315**	GRVI	− .469**	RSP	− .174**
SIA	− .531**	NDVI	− .533**	DEM	− .463**
SIB	− .531**	OSAVI	− .541**	CND	− .175**
SIT	.531**	RVI	− .534**		
SAIO	.525**	SAVI	− .550**		

**Significant *p* < 0.01; *Significant *p* < 0.05.

Among the raw bands of remote sensing, the correlations with soil EC were NIR (R = − 0.610), SWIR2 (R = 0.423), Red (R = 0.372), SWIR1 (R = 0.3), and Green (R = 0.246) in descending order. Salinity indices, as direct indicators in salinity monitoring^57^, showed good correlation with soil EC, and all nine selected salinity indices were significantly correlated with EC values, with correlation coefficients up to 0.531(SIA, SIB, SIT, SAIO are all salinity indices, which are different combinations of different waveforms), The correlation between vegetation index and soil EC values in descending order is, GARI (R = − 0.626), EVI (R = − 0.596), DVI (R = − 0.572), GDVI (R = − 0.541), OSAVI (R = − 0.541),RVI (R = − 0.534), NDVI (R = − 0.533), SAVI (R = − 0.550), CRSI (R = − 0.506), GRVI (− 0.469), GNDVI (R = − 0.468), it can be seen that the vegetation index is a good Indicator as an Indirect Indicator of salinity monitoring. Compared to NDVI, SAVI increases the vegetation signal and decreases the soil background, therefore, there is a strong correlation with soil EC (R = − 0.55), in addition, OSAVI has the same correlation as SAVI, but OSAVI avoids the complex calculation of soil baseline parameters. Among the topographic correlation factors, the higher correlation is with DEM (R = − 0.463), followed by CND (R = − 0.175), and finally RSP (R = − 0.174). The lower correlation between topography and Its Indices with EC Is explained by the overall flatness of the Werigan–Kuqa Oasis. Finally, the carbonate index CAEX correlated significantly (R = 0.612) with soil EC values, which were determined by the soil properties of the study area.

### Importance of selected environmental covariates

Different environmental factors have different predictive contributions to soil EC in predictive models, and not all environmental factors are significant variables in the modeling^58^, so it is necessary to rank the importance of environmental variables, and this study will rank the importance of features using each of the three models themselves and observe the differences in the contribution of variables in the three models.

**Figure 3:**
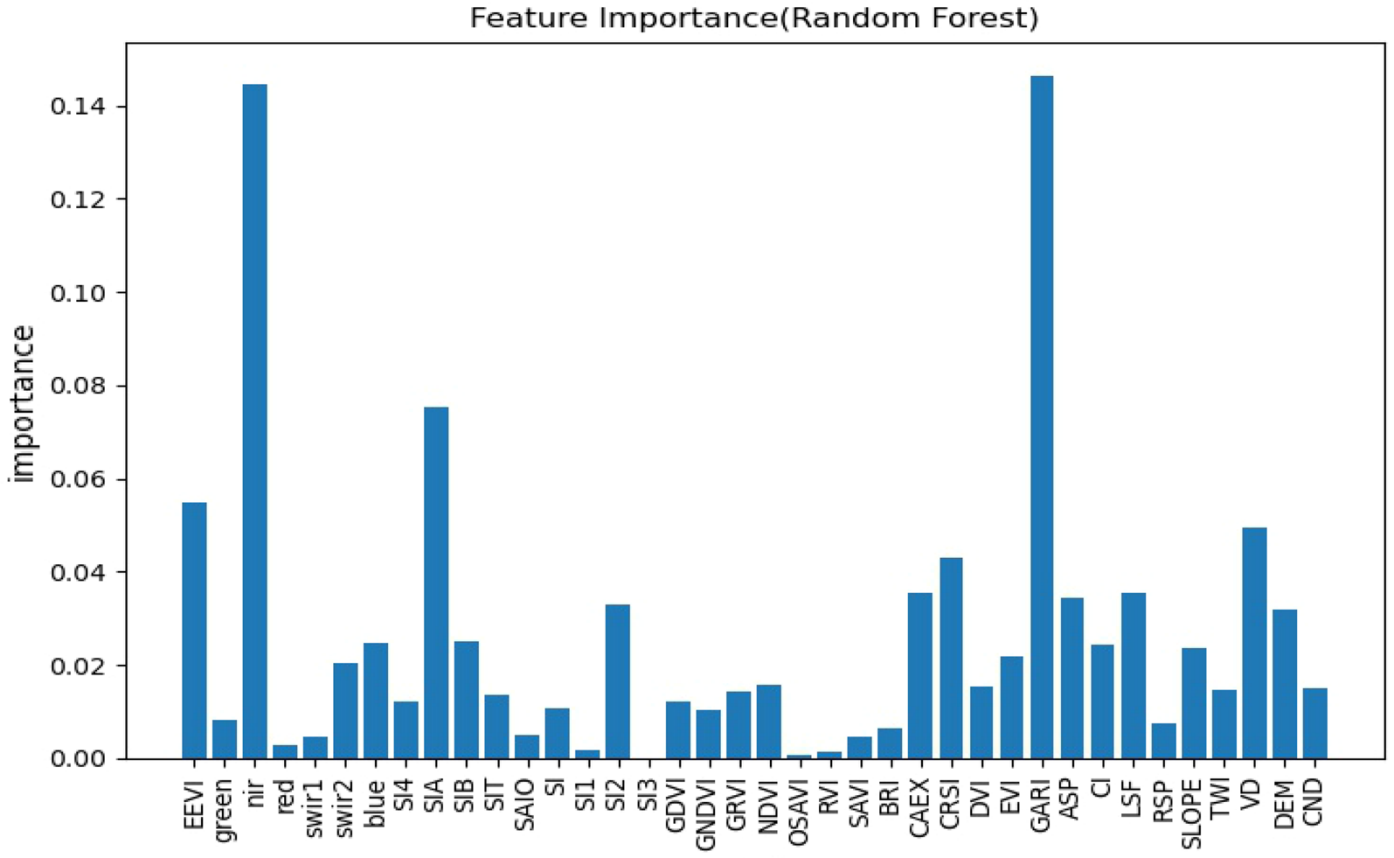
Characteristic importance diagram of RF.

**Figure 4:**
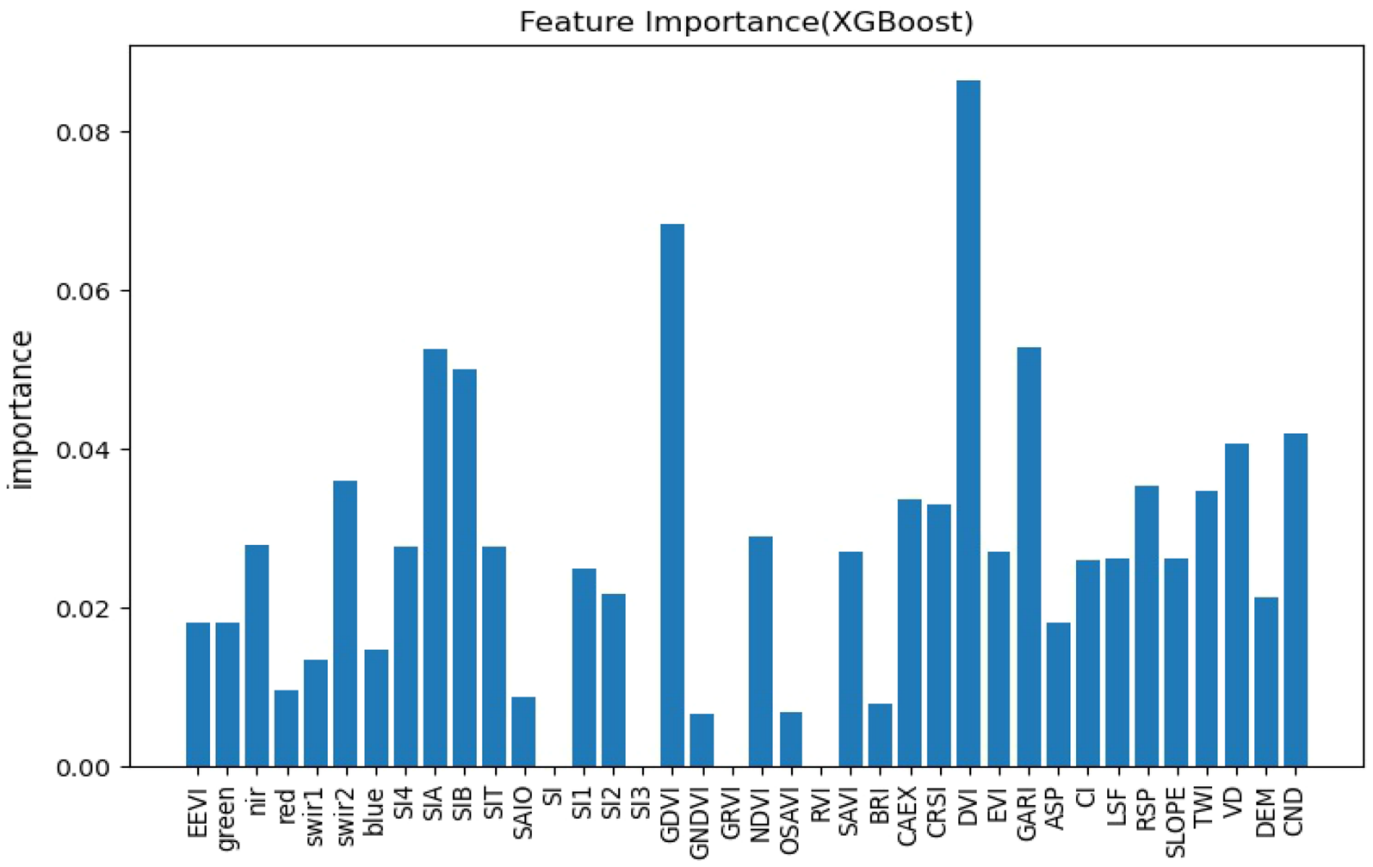
Characteristic importance diagram of XGBoost.

**Figure 5:**
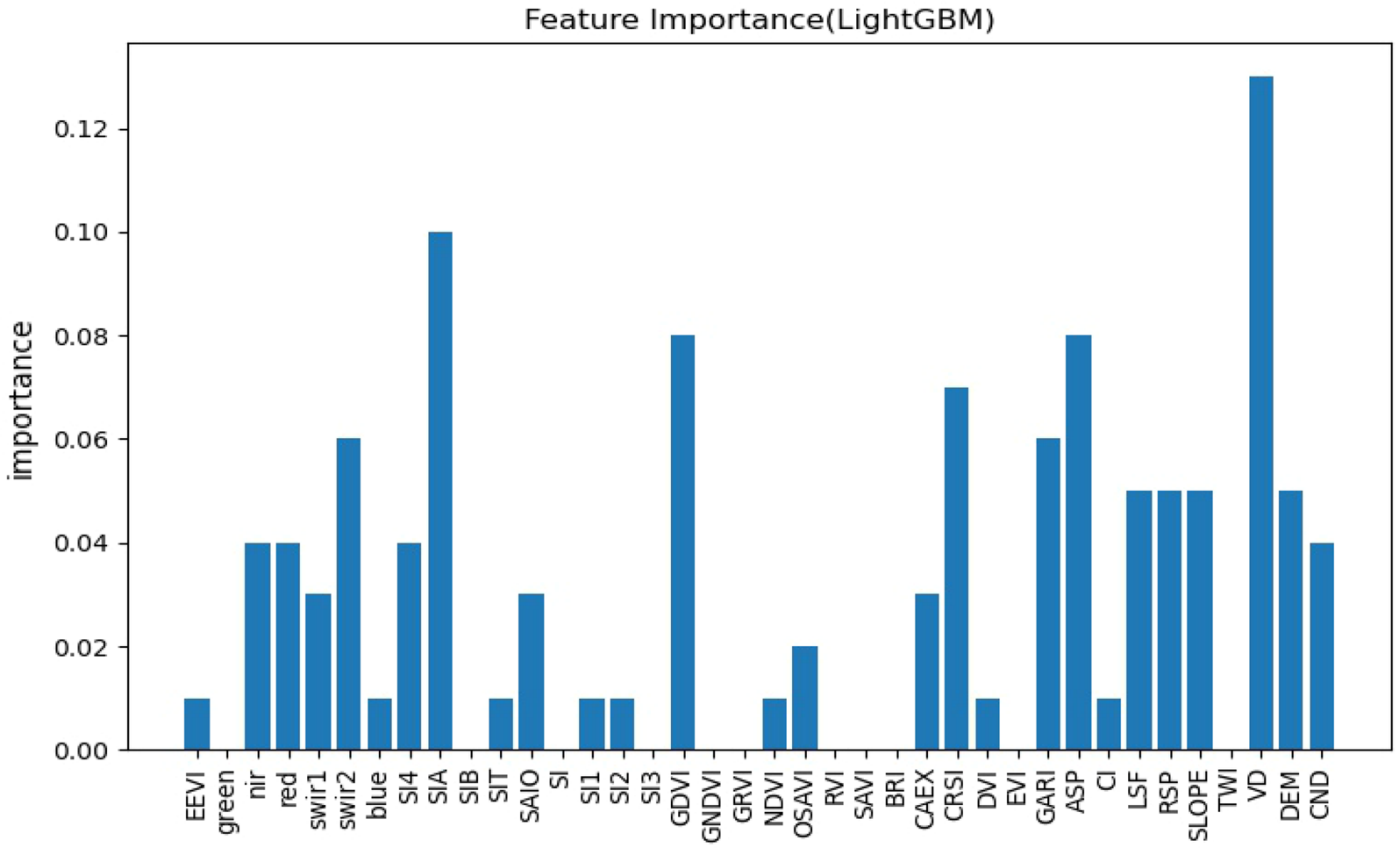
Characteristic importance diagram of LightGBM.

Figures 3, 4, 5 show the results of the three models for feature selection, the degree of contribution of the variables differed, but individual variables showed high contribution in all three models, and among the vegetation indices, most of them generally contributed well, with CRSI being the most stable and showing high contribution in all three models, in agreement with Scudiero et al.^34^ and Wu et al.^59^, GARI performed best among all environmental variables involved in RF. Remote sensing primitive bands are pivotal in the participation in modeling, in the study of related scholars, the relationship between each band and saline soils was analyzed in detail, the greater the salt in the soil, the higher the reflectance of all TM spectral bands^59^ and the spectral reflectance of CaCO_3_, CaSO_4_⋅2H_2_O, and gypsum sand were analyzed in the laboratory, they concluded that salt minerals can be detected when they are the main soil component^60^, among the primitive bands involved in modeling, the NIR band stands out, especially in the participation in the random forest modeling process, the contribution is second only to GARI. The salinity index stands out as a direct indicator in sparsely vegetated areas, and the SIA performed consistently in this study in terms of contribution across the three prediction models. The salinity index integrates most of the soil properties affected by salinity, and the salinity index is also very cost-effective for possible large-scale surveys to prevent soil salinity at the landscape scale^57^.

### Prediction accuracy

**Figure 6:**
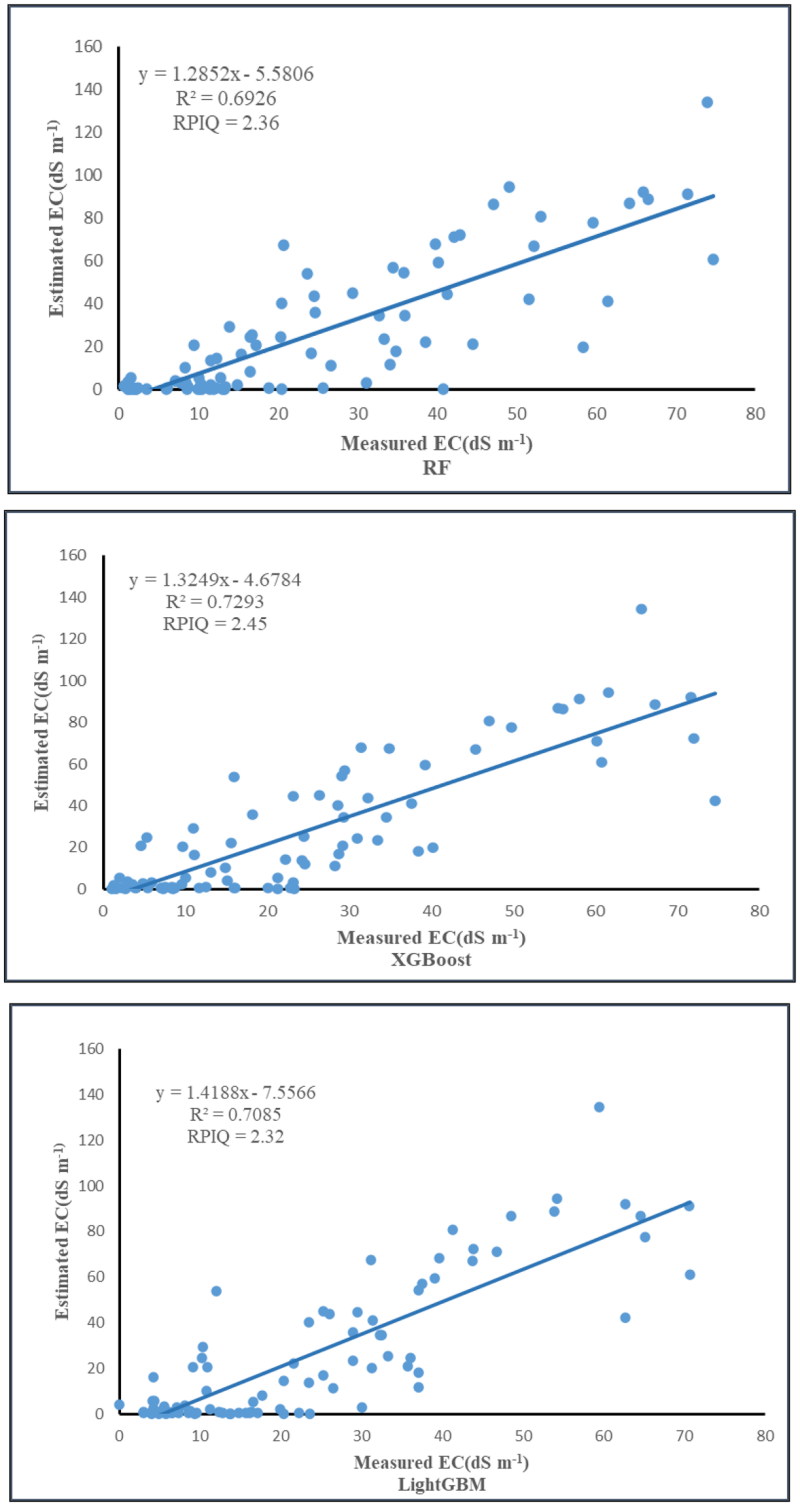
Measured and predicted regression analysis of the three models.

In this experiment, two approaches are used for model validation, the validation approach of slicing the dataset into training and test sets, and the cross-validation approach (Table 6, Fig. 6), and it was found that the R^2^ value of XGBoost was the highest among the three models in both the training and test sets, 0.84, 0.73, respectively, and the RMSE value was also the lowest in the training and test sets, 13.57 dS m^−1^, 17.62 dS m^−1^, respectively. The RPIQ value is also the highest, 3.32 in the training set and 2.45 in the test set. When RPIQ ≥ 2.2, it means that the model achieves excellent prediction, and compared with the performance of RF and LightGBM models in the test set (2.39 and 2.32, respectively), XGBoost has excellent prediction ability. Similarly, XGBoost has the lowest RMSE value of 19.9 dS m^−1^ for the three models after tenfold cross-validation. Therefore, XGBoost will be used as the optimal model for the digital mapping of the spatial distribution of salinity.

**Table 6:** The performance of each of the three models in the validation set and training set.

Algorithm	Calibration			Validation			Cross-validation	
	R^2^	RMSE	RPIQ	R^2^	RMSE	RPIQ	R^2^	RMSE
RF	0.82	14.3	3.15	0.69	18.19	2.39	0.6	20.29
XGBoost	0.84	13.57	3.32	0.73	17.62	2.45	0.6	19.9
LightGBM	0.71	18.57	2.43	0.64	18.58	2.32	0.57	20.1

### Spatial and temporal distribution characteristics and evolutionary trends of Salinization in 1996, 2006, 2017, and 2021

**Figure 7:**
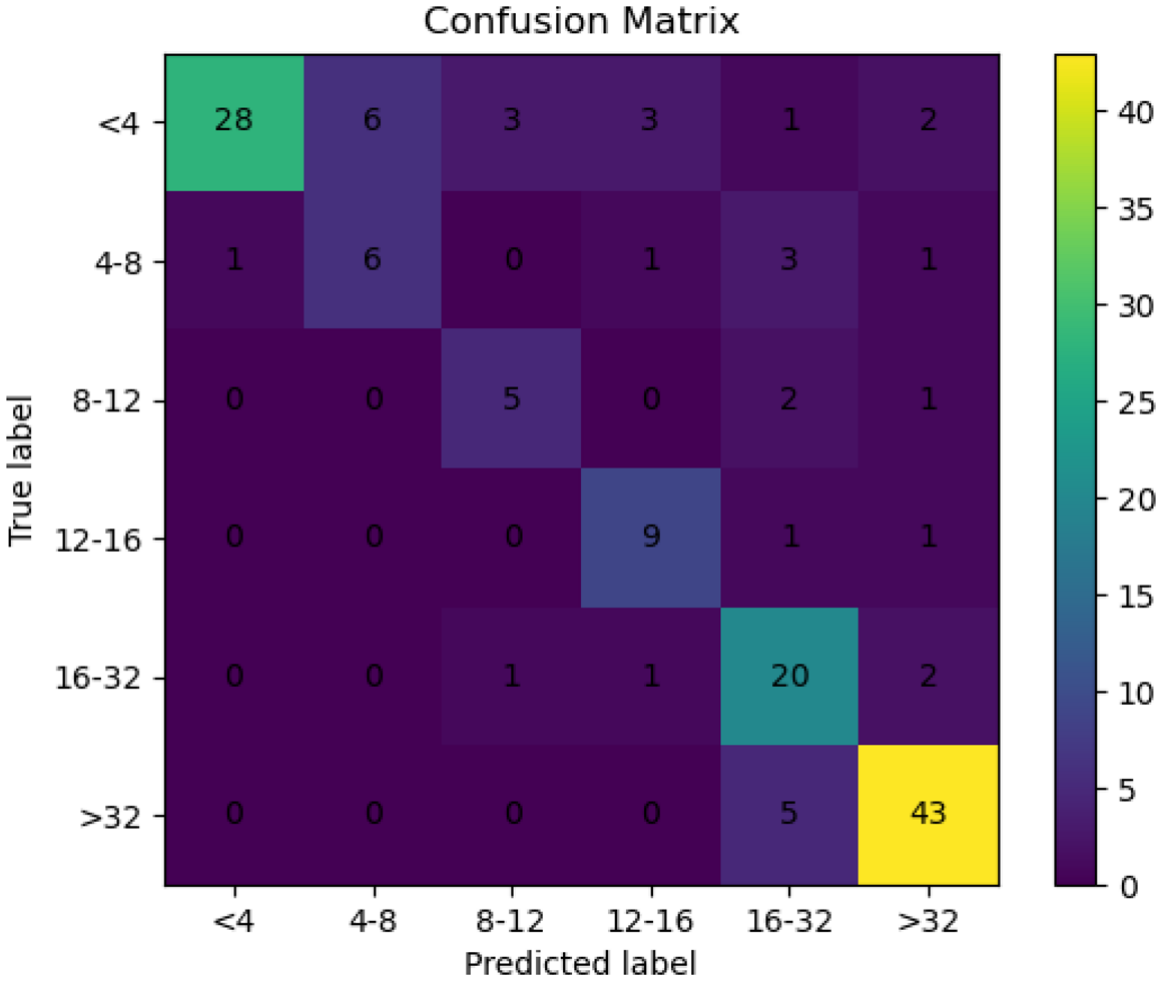
Confusion matrix verification.

In the research region, all soil samples were divided into six groups by the frequently used soil salinity classification method for further analysis and visualization^61^ (Table 7), and the spatial distribution of soil salinization in the Werigan–Kuqa Oasis on August 11, 1996, July 22, 2006, July 4, 2017, and July 15, 2021, were inverted using the selected optimal model and the corresponding optimal variables (Fig. 7). To further verify the accuracy of the salinity spatial distribution map after reclassification, this experiment used the 2017 and 2021 sample points as the validation set, and the accuracy was verified using the confusion matrix and kappa coefficient (Fig. 7), and the kappa coefficient was obtained as 0.71, which indicates that the salinity map has a high degree of consistency.

**Table 7:** Grades or classes of soil salinity.

Salinity constraint	EC(dS m^−1^)
Non-salinization	4 >
Mild salinization	4–8
Moderate	8–12
Heavy salinization	12–16
Extremely high	16–32
Saline soil	32 <

**Figure 8:**
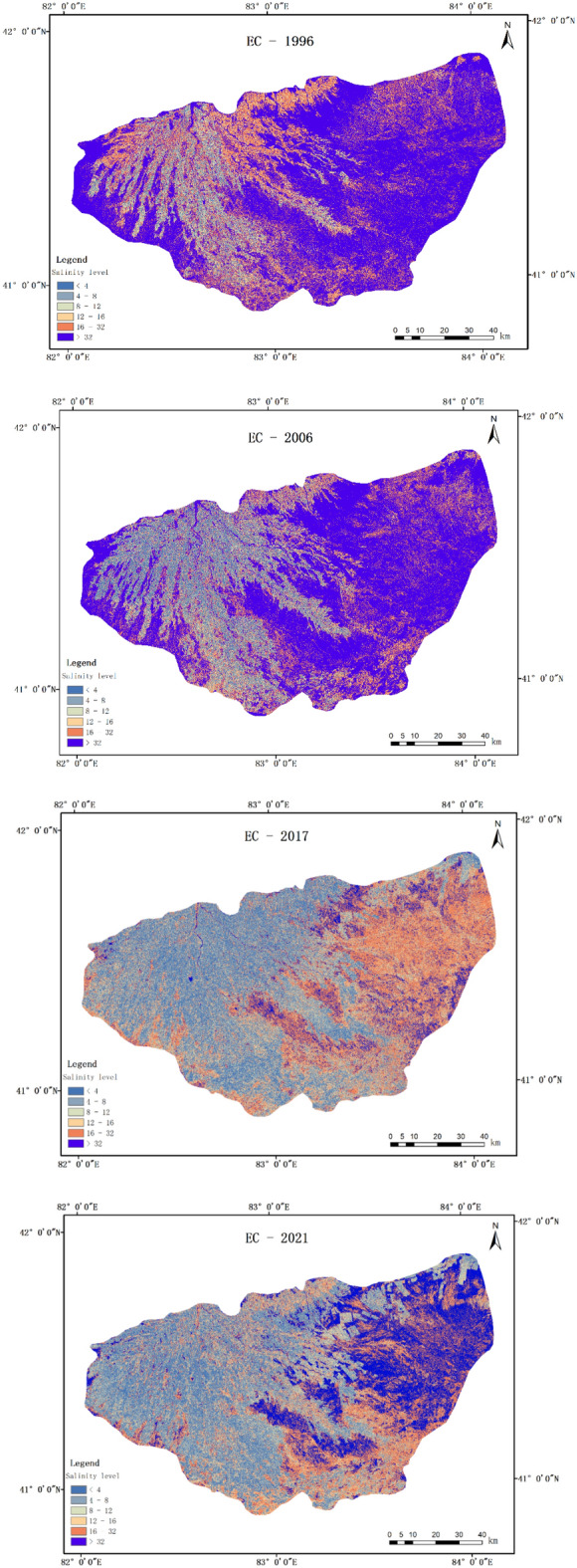
Spatial distribution of soil salinization in 1996, 2006, 2017 and 2021.

According to (Fig. 8), the spatial distribution of salinization in the Werigan–Kuqa Oasis shows a distribution characteristic of good in the west and north and severe in the east and south. The moderate and below salinization in the Werigan–Kuqa Oasis is distributed in the west and north of the Werigan–Kuqa Oasis, an oasis area with good irrigation conditions (Fig. 1), where the main feature type is arable land, the terrain is relatively high, not easily waterlogged, and the vegetation cover is relatively high. With the expansion of the spatial extent of arable land, light salinization and below also show a corresponding radial change to the south, southwest, and southeast, and become more continuous spatially. By 2021, on the western and southern edges of the Werigan–Kuqa Oasis, very heavy salinization has been transformed into light salinization, in the eastern and northeastern regions, spatially discontinuous new arable land emerged, so that mild salinization also took the form of sporadic spatial distribution.

Severe and very severe salinization was mainly distributed in the northern part of the Werigan–Kuqa Oasis in 1996, and by 2006, salinization in the region improved and gradually shifted to the east and south, developing to the southeast by 2021. The development trend of severe and very severe salinization over 25 years is closely related to the low southeast and high northwest topography of the Werigan–Kuqa Oasis (Fig. 1).

The most pronounced spatial distribution and evolutionary characteristics of saline soils with the highest degree of salinization were mainly distributed in the southwestern edge of the Werigan–Kuqa Oasis and most of the desert areas in the east in 1996, shifting to classes such as severe and very severe in 2017, and improving significantly by 2021, especially in the eastern desert areas. Relying on years of field surveys, it was found that sparse salt vegetation grows in the eastern part of the Werigan–Kuqa Oasis, while the southeastern part of the area is sparsely forested. As a result of enhanced vegetation protection efforts in the eastern area, the vegetation cover has increased significantly and, therefore, the evaporation of surface water has decreased accordingly, reducing the rate of salt accumulation on the surface.

### Change in area of salinization at different levels

**Figure 9:**
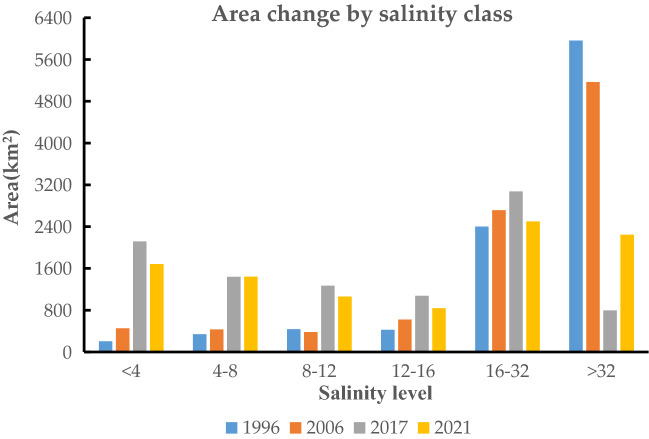
Trends in the area of different levels of salinization.

As shown in (Fig. 9), the non-salinized area of the Werigan–Kuqa Oasis is 198.25 km^2^ in 1996 and 1682.47 km^2^ in 2021, an increase of 748.6%; Mild salinization was 346.78 km^2^ in 1996 and increased year by year since then to 1441.29 km^2^ in 2021, an increase of 315.6% compared to 1996; Moderate salinization remained stable from 1996 to 2006 and increased substantially by 2017 to 1062.26 km^2^ by 2021, an increase of 134.8% compared to 1996; Heavy salinization was 431.26 km^2^ in 1996 and 838.132 km^2^ in 2021; Very heavy salinization remains relatively stable from 1996 to 2021, with an area of 2498.74 km^2^ by 2021; The area of saline soil was 5708.77 km^2^ in 1996, then declined to 5168.7 km^2^ in 2006, followed by a greater decline to 794.48 km^2^ in 2017 and 2246.87 km^2^ in 2021, a decrease of 60.6% compared to 1996. Based on the results of the above statistical analysis: during the last 25 years, the non-salinized, lightly salinized, and moderately salinized areas increased more, the saline soil area decreased more, and the heavy and very heavy salinization changed less and remained stable, so there was an improvement of soil salinization in the Werigan–Kuqa Oasis.

## Discussion

### Long-time series of salinity monitoring

Various multispectral sensors rely on the spectral reflectance properties of the ground for ground monitoring^62^, and the spectral reflectance varies for different levels of salinity, often with a white salt crust attached to the ground surface in highly saline areas. The higher the salinization, the higher the spectral reflectance of each band will increase accordingly^13^, therefore, it becomes possible to monitor salinization using raw bands or derived spectral indices of remote sensing. In previous studies on salinity monitoring, the choice of environmental variables varied, such as direct use of salinity indices for estimating soil salinity^63^, indirect estimation of soil salinity using vegetation indices^64^, or combining multiple environmental variables and grouping them to predict comparisons^58^.

The objective of this study is to map the spatial distribution of salinization in the Werigan–Kuqa Oasis in different years and analyze the changing trend of salinization area in different grades. Therefore, remote sensing data that can match the sampling time in different years are selected, and a stable soil EC prediction model is established based on the extraction of environmental variables from remote sensing images, which makes it possible to accomplish the goal of salinization spatial distribution mapping realistically and accurately and provide data reference for salinization management and water resources management. The earliest data collection in this study area began in 2006, so in this modeling, sample data from 2006, 2017, 2018, and 2021 were ensemble for modeling, making full use of the available laboratory data. This study utilizes the Google Earth Engine platform for fast online computational processing. Therefore, the remote sensing cloud platform presented by Google Earth Engine is an excellent option for environmental monitoring research that uses lengthy time series of remote sensing data.

### Spatial and temporal evolutionary characteristics of salinization

The distribution of saline salinization in the Werigan–Kuqa Oasis shows distinct regional characteristics. In the southeast and east of the Werigan–Kuqa Oasis, which is the most affected area by salinization, salinization of very severe and higher grades is distributed, and the spatial and temporal evolution characteristics are obvious. The low elevation compared to other areas of the Werigan–Kuqa Oasis (Fig. 1D) makes it possible to distribute high concentrations of salts in this area^40^. After years of field investigation and sampling, seasonal floods often gather in this area, and according to Ding and Yu^4^, it was found that the salts accumulated on the surface of the area do not drain outward, which makes it more difficult to manage salinization. In addition, the area is dominated by sandy soils, and during the dry season, salts are easily deposited on the surface after water evaporation^4^. During the 25 years, salinization in the eastern part of the Werigan–Kuqa Oasis has improved significantly because the local government has strengthened the vegetation protection of the desert, and built alkali drainage canals in the sparsely vegetated areas of the desert to reduce seasonal waterlogging to a certain extent, and strictly monitored overgrazing practices, so that the vegetation coverage and the area covered by the area have gradually increased, and therefore the area of very heavy salinization in the area has decreased in recent years.

In the southeast of the Werigan–Kuqa Oasis fringe area, salinization of severe and higher grades is distributed and has not improved significantly in individual areas during the last 25 years, which is since the economy of the study area is dominated by irrigated agriculture and surface irrigation is a common irrigation method, and the salts in the soil inside the Werigan–Kuqa Oasis are transported to the downstream through surface irrigation water, which deposits salts on the downstream surface and eventually intensifies the formation of salinization This is the reason why salinization is higher at the edge of the oasis than in the interior of the oasis^4^.

The salinization of moderate and lower grades is distributed in the interior of the oasis. Since the economy of the study area is based on irrigated agriculture, especially in the western and southwestern regions of the study area, which are more dependent on this economic activity, the formation of mild salinization in the region is strongly related to agricultural irrigation, while the irrigation of the regional arable land is gradually changing from the previous surface irrigation to drip irrigation, which may aggravate salinization in the region. The spatial and temporal evolution of salinization within the oasis is also more pronounced during the 25 years, due to the expansion of the arable land area, which increases significantly by 2021 compared to 1996, especially in the southwest and northeast of the study area, and therefore, the salinization grade changes accordingly, from severe and above grade to moderate and below, and to ensure healthy crop survival, before planting The land is drained of alkali to ensure healthy crop survival. In addition, the salinization of arable land areas tends to be consistent, and the area of salinization of heavy and above grades is reduced and fragmented, because the local government has been carrying out comprehensive land improvement work, leveling dry land and barren land; renovating and reinforcing branch canals and field branch; building rural field roads less than 4.5 m, serving production and travel, especially since 2018, the local government has carried out the construction of high-standard farmland, making the land more flat and contiguous, with better agricultural facilities, more fertile land and better disaster resistance. The results of the study show that human activities are the key factors affecting the aggravation and management of salinization^58^, and the key lies in whether humans destroy or protect land and water resources, and as the core area of the Belt and Road, it should focus on the protection of the ecological environment, and its starting point should be the management of salinization in arid areas. The irrational use of water resources is related to the salinity of the soil^65^, so in the future, we should discuss the planting pattern of the Werigan–Kuqa Oasis and a more economical and efficient irrigation method. It is gratifying to note that the government has in recent years become more disciplined in water resources management, such as the implementation of the river chief system, which strictly regulates the reckless diversion of rivers; the implementation of the water station chief system in irrigation areas, which provides more precise and efficient control of irrigation water resources; and the implementation of the forest chief system, which increases the protection of forest land. Through these measures, the salinization of the Werigan–Kuqa Oasis has been improved.

## Conclusions

This study uses multi-year field collection data and multi-source data with the help of the ensemble learning method and Google Earth Engine cloud platform to complete the digital mapping of salinity spatial distribution in 1996, 2006, 2017, and 2021, analyze the spatial and temporal evolution characteristics and driving factors of salinity in Werigan–Kuqa Oasis, and draw the following conclusions:

1. Among the three ensemble learning models, RF, XGBoost, and LightGBM, XGBoost had an RMSE of 17.62 dS m^−1^, R^2^ of 0.73, and RPIQ of 2.45 in the test set, which had higher prediction accuracy compared with the other two models, and more accurate salinization distribution maps were obtained using XGBoost.
2. The salinization in the study area generally shows the distribution characteristics of good in the west and north and severe in the east and south. The moderate and below salinization is distributed in the oasis areas with good irrigation conditions and smooth drainage. And severe and above salinization is mainly distributed in the desert areas in the east and southeast.
3. The spatial and temporal variation of salinization in the study area has changed significantly in the last 25 years, with non-salinization and light salinization expanding in the east and southwest spatial distribution with the increase of arable land area and effective remediation planning of arable land. The distribution area of salinization of severe and above grades has shrunk more significantly.

## Data Availability

The datasets generated and analyzed during the current study are available from the corresponding author upon reasonable request.
